# Can sharing auditors with customers improve suppliers digital transformation?

**DOI:** 10.3389/fpsyg.2024.1336653

**Published:** 2024-06-28

**Authors:** Xiaohui Liu, Yan Chen

**Affiliations:** School of Accountancy, Shandong University of Finance and Economics, Jinan, China

**Keywords:** shared auditor, digital transformation, supply chain, innovation, suppliers

## Abstract

**Introduction:**

Promoting enterprises’ digital transformation is fundamental to implementing the digital China strategy and realizing high-quality development.

**Methods:**

Taking China’s A-share listed companies from 2007 to 2021 as a research sample, this study examines the impact of sharing auditors with customers on the digital transformation of suppliers.

**Results:**

The results show that sharing auditors with customers can promote suppliers’ digital transformation, and this promotion effect is stronger among suppliers with weaker bargaining power, lower media attention, and higher auditor industry expertise. In terms of the mechanism of action, sharing auditors with customers can help strengthen the supplier’s supervision, alleviate suppliers’ financing constraints, and thus facilitate their digital transformation. Further research finds that when suppliers share auditors with their customers, suppliers’ digital transformation helps promote innovation.

**Discussion:**

The research conclusion provides effective empirical evidence for alleviating the dilemma of digital transformation of enterprises.

## 1 Introduction

With the slowdown of global economic growth, the international market is showing a weak demand trend, and overcapacity has become a widespread phenomenon. Especially for China, rapid industrialization and economic development have caused China’s overcapacity problem to show Chinese characteristics such as large-scale and long-term. The imbalance between supply and demand structures, such as high product inventory, restricts the development of Chinese enterprises. In this context, it is important for suppliers to grasp customer needs and maintain long-term and stable customer relationships. Sharing auditors with customers is an effective way to understand customer needs and maintain stable customer resources by establishing a social network between suppliers, enterprises, and customers. Customers are important stakeholders who “rise and fall together” with suppliers, and customer-related information, such as customer needs, is a key factor suppliers consider when making investment decisions. As a result, sharing auditors with customers can promote the flow of information between suppliers and customers, which is conducive to establishing interdependent, mutually beneficial, and win-win cooperative relations, which in turn has a significant impact on the investment decisions of suppliers.

At present, the digital economy is becoming an important driving force for global economic growth. As an important micro subject of the market economy, enterprises are the core carrier on which the development of the digital economy depends, and promoting the digital transformation of enterprises is the key engine for the growth of the digital economy. However, in reality, some enterprises lack the motivation and ability for digital transformation due to the large demand for transformation funds and the uncertainty of transformation effects. Sharing auditors with clients can significantly enhance the motivation and ability of suppliers to implement digital transformation. Specifically, because digital transformation can help suppliers form advantages that are difficult to imitate externally (Tong et al., 2024), it can help suppliers win customer trust and favor. Suppose the digital transformation of the supplier is effective, the shared auditor can better communicate this information to the stakeholders, which is conducive to the supplier attracting potential customers and maintaining high customer stickiness. Based on this, this study will deeply explore the impact of the shared auditors on the digital transformation of suppliers and their internal mechanism for sharing with customers and try to find a new way to solve the dilemma of enterprise digital transformation.

The research contributions of this study are as follows: First, it explores the impact of sharing auditors with customers on the digital transformation of suppliers from the perspective of the supply chain and provides new insights into the motivation of enterprise digital transformation. Although the existing literature examines the influencing factors of digital transformation, from the internal and external factors of enterprises, it ignores the role of shared auditors in driving the digital transformation of enterprises. In fact, sharing auditors with clients can embed an important source of power for the digital transformation of the vendor enterprise. By examining the impact of shared auditors with customers on the digital transformation of suppliers, this study not only helps to understand the positive governance role of shared auditors but also provides a logical explanation for the causes of enterprise digital transformation and provides new ideas for alleviating the problems of enterprise digital transformation. Second, this study analyzes the heterogeneity based on the bargaining power of suppliers, the media attention of suppliers, and the industry expertise of auditors, which provides a more comprehensive perspective for the study of the relationship between shared auditors and the digital transformation of suppliers, which is of enlightening significance for how to solve the problem of enterprise digital transformation more efficiently. The findings of the study contribute to a better understanding of the positive impact of sharing auditors with clients on the digital transformation of suppliers.

## 2 Literature review

### 2.1 Research on shared auditors between suppliers and customers

Existing literature argues that supply chains are both economic and information chains, and when suppliers share auditors with their customers, the auditors obtain more information about the suppliers and their customers in the course of performing audit procedures, generating knowledge spillovers (Cai et al., 2018), which in turn have a significant impact on suppliers’ behavior.

From the perspective of knowledge spillovers, when suppliers share the same auditor with their customers, the auditor can mutually corroborate information related to round-trip sales and after-sale repurchases between the suppliers and their customers, reduce sales-related financial restatements, improve financial statement auditing quality (Chen et al., 2012; Yang et al., 2015), and reduce surplus forecasting bias (Cai et al., 2018). As an important service organization for enterprises, shared auditors act as information intermediaries in the supply chain (Hu et al., 2022a,b). Shared auditors obtain more detailed and cutting-edge information about the future cash flow status, production and operation plans, and potential risks on both sides of the supply chain by embedding themselves into the relationship network between suppliers and their customers (Yang et al., 2015; Cai et al., 2019), alleviate the problem of information asymmetry in the supply chain, which in turn affects the increase of enterprises’ relationship-specific investment (Dhaliwal et al., 2017; Hu et al., 2022b), improve financial statement comparability (Jiu et al., 2020), and increase the degree of enterprises’ tax avoidance (Hu et al., 2022b).

### 2.2 Research on factors influencing the enterprise digital transformation

In essence, enterprise digital transformation integrates cutting-edge digital technologies such as blockchain and big data into business, strategy, and management to enhance user experience, streamline operational processes, and develop new business models (Warner and Wäger, 2019). Due to the uneven quality of digital business service providers, such as big data and cloud computing in the market, taking cloud computing as an example, some cloud service providers have problems such as imperfect data backup, imperfect key management strategies, and insufficient business security risk control capabilities, which can easily lead to user data leakage.^1^ Concerns about data breaches and uncertain results of digital transformation have led some enterprises to become confused about whether to carry out digital transformation. However, it is clear that China has enjoyed the digital dividend in the process of developing the digital economy (Liu et al., 2021); the atmosphere of digital transformation is becoming stronger, more and more enterprises are joining the wave of digital transformation, and the academic community has begun to discuss what factors will promote the digital transformation of enterprises.

Existing research shows that the factors influencing the digital transformation of enterprises include internal and external factors. In terms of internal influencing factors, Li et al. (2018) took cross-border e-commerce SMEs as a case study and proposed that the upgrading of organizational capabilities is an important prerequisite for achieving transformation. Warner and Wäger (2019) argue that digital dynamic capabilities, consisting of digital perception, digital capture, and digital reconstruction, are prerequisites for enterprises to gain a competitive position in the digital economy. Porfírio et al. (2021) used the fs QCA method to conclude that enterprise characteristics (such as size) and management characteristics (such as leadership style) are important factors influencing digital transformation. In addition, employees’ digital skills (Kozanoglu and Abedin, 2021), CEOs with overseas backgrounds (Hu D. M. et al., 2022), and foreign shareholders (Wang, 2023) are all internal factors influencing digital transformation.

From the perspective of external drivers, some scholars believe it is particularly important to examine the causes of enterprise digital transformation from the institutional level (Lai and Yue, 2022). Lai and Yue (2022) used the “National Smart City Pilot” to design a quasi-natural experiment and found that the construction of smart cities can solve the dilemma of financial constraints and talent constraints for digital transformation, thereby promoting the digital transformation of enterprises. Tang et al. (2022) found that interest rate liberalization can empower the digital transformation of enterprises by alleviating their financing difficulties and reducing corporate leverage. Some research also focuses on the impact of external stakeholders, especially customers, on digital transformation. Zhang and Ma (2022) found that dependent customer relationships can worsen the financial status of enterprises and reduce their ability to obtain resources, thereby hindering the digital transformation of enterprises, while peer-to-peer customer relationships can improve the financial status and governance of enterprises, thereby promoting the digital transformation of enterprises. Wang et al. (2024) found that enterprises that are geographically distant from large customers are more similar to the digital transformation orientation of large customers.

It can be seen that as a change in line with national policies and economic practice orientation, the digital transformation of enterprises has received extensive attention from the academic community. However, from a practical point of view, there are still many obstacles in the process of enterprise digital transformation, and “will not transform” and “dare not transform”^2^ have become obstacles in the practice of enterprise digital transformation. For this, in March 2020, to accelerate the pace of enterprise digital transformation, the National Development and Reform Commission (NDRC) provided two dimensions of force and policy measures; one is the digital transformation partner action to solve the problem of “will not transform,” and the other is the construction of a digital supply chain to alleviate the dilemma of “dare not transform.” The combination of measures seems to indicate the important role of the supply chain in digital transformation. Shared auditors play a key role in information transfer and resource integration in the supply chain, so it is necessary to clarify the impact and mechanism of sharing auditors with customers on the digital transformation of suppliers.

## 3 Theoretical analysis and research hypotheses

### 3.1 Shared auditors and supplier digital transformation

The existing literature suggests that the Motivation-Opportunity-Ability (MOA) theory provides a reasonable theoretical basis for explaining enterprise behavior (Gruen et al., 2005). Among them, motivation refers to the subjective driving force that leads an enterprise to adopt a certain behavior, ability refers to the resources necessary to adopt a certain behavior, and opportunity refers to the objective environmental factors that are conducive to motivating the enterprise’s behavior but are not controlled by the enterprise (Auh et al., 2007; Mao et al., 2024). The Chinese government has issued several strong policies to support enterprises’ digital transformation; that is, Chinese enterprises already have favorable opportunities for digital transformation (Mao et al., 2024), but how to enhance the motivation and ability of enterprise digital transformation has become the key to promoting enterprises digital transformation. Sharing auditors with customers is an effective way to enhance the motivation and ability of suppliers’ digital transformation.

From the perspective of motivation, according to the theory of organizational behavior, the willingness to act is the decisive factor that constitutes organizational behavior (Davis, 1989); that is, the will drives the behavior to occur. As a systematic innovation activity, digital transformation has the characteristics of a difficult transformation, a long transformation cycle, and an uncertain transformation effect. Compared with auditors who only provide audit services to suppliers, sharing auditors with customers can help stakeholders strengthen the supervision and restraint of managers’ behavior and enhance managers’ willingness to carry out the digital transformation that is conducive to the long-term development of enterprises. In particular, when a client undergoes digital transformation to meet the client’s needs for high-quality accounting information and digital business quality assurance in the process of digital operation, the accounting enterprise may assign a technical auditor to the client (Yao, 2024). When the shared auditor provides services to the supplier, the auditor can use new technologies such as machine learning to increase the interaction and contact with the supplier and obtain more real-time data of the supplier, which is conducive to restricting the behavior of the supplier managers, prompting the supplier to increase long-term investment projects, and enhancing the willingness of the supplier to digital transformation.

From the perspective of capability, as a new innovation strategy, the implementation of digital transformation is inseparable from long-term and high-cost investment, and a large amount of financial support is required for the large-scale application of digital technology, the construction of digital infrastructure, and the update and iteration of equipment. The existing literature shows that when a supplier shares an auditor with a customer, a social network can be formed between the auditor, the supplier, and their customers (Zheng and Zhu, 2021), which can help the supplier accurately assess the customer’s product demand, effectively reduce the uncertainty of product demand, reduce inventory, and improve transaction efficiency to ensure sufficient liquidity of the enterprise and provide financial support for digital transformation (Li and Li, 2023). Moreover, sharing the auditor with the client can improve the audit quality of the financial statements of the supplier (Chen et al., 2012; Yang et al., 2015). Higher audit quality means that the financial reports of suppliers are more authentic and reliable, which reduces the additional financing costs that suppliers need to pay due to information asymmetry to a certain extent, thereby alleviating the financing constraints of suppliers and promoting the digital transformation of suppliers.

**Figure 1:**
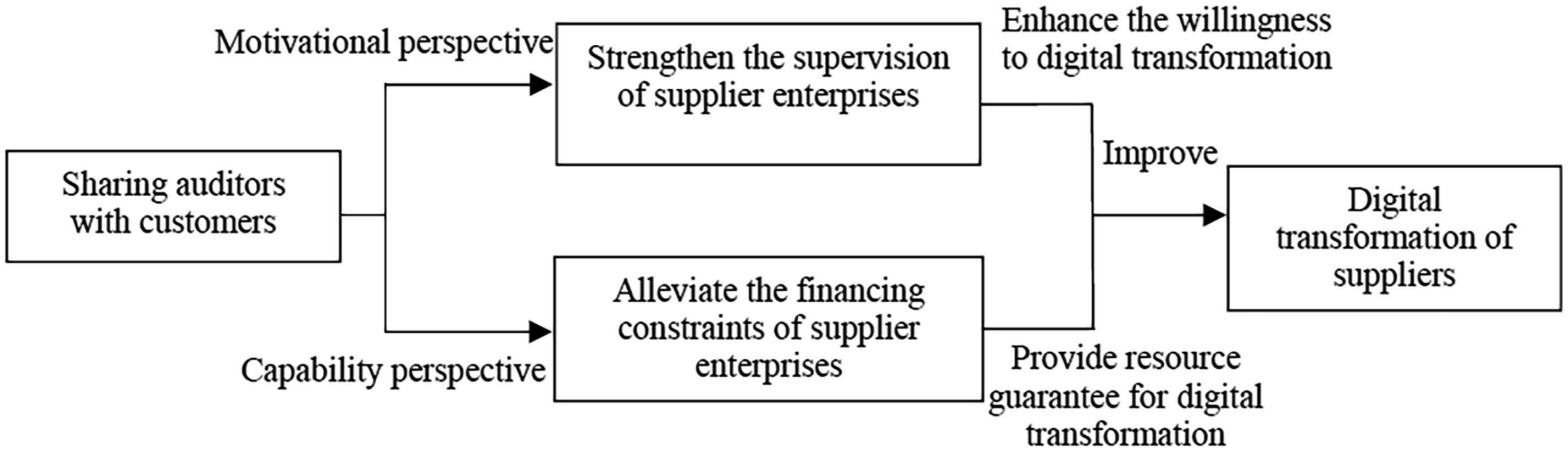
Mechanisms for sharing auditors with customers to influence the digital transformation of suppliers.

In summary, from the perspective of motivation, sharing auditors with customers can help strengthen the supervision of suppliers and enhance the subjective willingness of suppliers to transform digitally. From a capability perspective, sharing auditors with customers can help alleviate financing constraints and provide the necessary resources for the digital transformation of suppliers (the influence mechanism of sharing auditors with customers on the digital transformation of suppliers is shown in Figure 1).

Based on the above analysis, we propose the following hypothesis:

*Hypothesis 1:* Sharing auditors with customers can improve the digital transformation of suppliers.

### 3.2 Shared auditors, bargaining power, and suppliers’ digital transformation

The bargaining power of suppliers is one of the crucial issues in supply chain research and an important influence on suppliers’ decision-making, and the bargaining power of suppliers may affect the effect of sharing auditors with customers on the digital transformation of suppliers. Suppliers with weak bargaining power are in a weak position in the game of supply chain relationship transactions (Porter, 1979; Panousi and Papanikolaou, 2012), and it is usually difficult for them to obtain customers’ accurate demand information through private conversations with customers, resulting in suppliers being in an information disadvantageous position (Chiu et al., 2019). Meanwhile, suppliers with weaker bargaining power usually face resource constraints, and their ability to obtain additional information through consulting organizations is weaker (Mao et al., 2023); thus, such suppliers are more dependent on shared auditors, and they are more dependent on shared auditors to verify the truthfulness and accuracy of the information provided by customers. It can be hypothesized that sharing auditors with customers has a greater impact on the digital transformation of suppliers with weaker bargaining power. Therefore, we propose the following hypothesis:

*Hypothesis 2:* Sharing auditors with customers is more helpful in improving the digital transformation of suppliers with less bargaining power than suppliers with more bargaining power.

### 3.3 Shared auditors, media attention, and suppliers’ digital transformation

The media is a third-party mechanism in the capital market, which plays an important role in disseminating information and supervising public opinion. Higher media attention means that suppliers’ investment decision-making behavior is exposed to the public eye, and corporate strategies that merely pursue short-term performance while neglecting long-term corporate development will be more easily identified and infinitely amplified by external stakeholders. Conversely, lower media attention may lead to opportunistic behavior by managers due to a lack of external oversight, hindering suppliers’ digital transformation. In this case, if suppliers share the same auditor with their customers, the auditor will be able to corroborate key information such as round-trip sales and after-sale repurchases between the suppliers and their customers, and the information manipulation and opportunistic behaviors of the suppliers will be more easily detected by the auditor, thus increasing the cost of opportunistic behaviors of the suppliers and helping to incentivize the management to carry out digital transformation with value-added effects. This will increase the cost of the opportunistic behavior of suppliers and help motivate management to carry out digital transformation with “value-added” effects. In summary, suppliers with low media attention rely more on shared auditors to increase the cost of opportunistic behavior and motivate suppliers to actively engage in digital transformation, suggesting that shared auditors can serve as an effective alternative to other forms of external oversight.

*Hypothesis 3:* Sharing auditors with customers is more helpful for digital transformation in suppliers with lower media attention than in suppliers with higher media attention.

### 3.4 Shared auditors, auditor industry expertise, and suppliers’ digital transformation

Because implementing a digitalization strategy is characterized by high costs and risks (Hu D. M. et al., 2022), managers have strong incentives to seek high-quality information to reduce the potential uncertainty of digital transformation. Compared to managers, auditors with industry expertise can objectively judge industry prospects and accurately grasp potential investment opportunities based on their rich knowledge and experience (Francis and Yu, 2009). As a result, auditors with industry expertise can effectively supplement the information environment of managers and help them acquire valuable incremental information, thus reducing uncertainty during the digital transformation and increasing managers’ motivation to carry out digital transformation. Meanwhile, based on both reputation and professional competence, auditors with industry expertise are more inclined to monitor and inhibit the opportunistic behavior of enterprises, prompting enterprises to pay more attention to digital transformation that contributes to the long-term development of the enterprise.

*Hypothesis 4:* Sharing auditors with industry expertise with customers is more conducive to the digital transformation of suppliers.

## 4 Research design

### 4.1 Sample selection

In this study, we take China’s A-share listed enterprises from 2007 to 2021 as sample and treat the initial sample as follows: (1) excluding samples whose top five customers of listed enterprises are non-listed enterprises and those with missing customer information; (2) excluding samples with abnormal trading status, such as ST, *ST, etc.; (3) excluding samples of the financial industry where there are peculiarities in the accounting; and (4) eliminating samples with missing data. In this study, the digital transformation data and the top five customers of listed enterprises are from the CNRDS database, and the rest of the data are mainly from the CSMAR database. To reduce the influence of extreme values, all continuous variables are winsorized at the 1% level.

### 4.2 Definition of variables

#### 4.2.1 Enterprise digital transformation

Referring to the research of Lee et al. (2024), the digital transformation of enterprises is measured from two dimensions: digital transformation keyword frequency (*DT*) and digital transformation investment (*DI*). On the one hand, referring to Wu et al. (2021) and Shang et al. (2023), this study uses the frequency of keywords related to the digital transformation of enterprises in the enterprise’s annual report to measure *DT*. The keywords of enterprise digital transformation can be divided into two levels, namely “underlying technology application” and “practical application of technology.” The digitization keyword frequencies of the above two levels are summed up, and the natural logarithm is taken after adding 1 to get the indicators for measuring *DT*. On the other hand, the specific measurement method of *DI* refers to the research of Wu and Yang (2024), which is measured by the ratio of the amount of hardware and software investment in enterprise digital transformation to the total assets of enterprises. Specifically, digital transformation software investment is measured by software and information systems investment in intangible asset items in the annual report of the study sample; digital transformation hardware investment is measured by the investment in electronic equipment and computers in the fixed asset items in the annual report of the study sample.

#### 4.2.2 Shared auditors

Referring to Dhaliwal et al. (2017), we define *Comaud* as a dummy variable that equals 1 if a supplier shares its audit enterprise with at least one of its top five customers in the current year and 0 otherwise.

#### 4.2.3 Control variables

The control variables are as follows: (1) enterprise size (Size); (2) enterprise age (Age); (3) shareholding ratio of the top 10 shareholders (Top10); (4) gearing ratio (Lev); (5) return on assets (Roa); (6) operating cash flow (Cfo) are selected; (7) the accounting enterprise size (Big4); and (8) audit opinion (Opinion). Specific variables are defined in Table 1.

**Table 1:** Definitions of key variables.

Nature of variables	Variable name	Variable symbol	Variable definition
Dependent variable	Digital transformation keyword frequency	*DT*	Add 1 to the natural logarithm of the frequency of the keyword “digital transformation” in the enterprise’s annual report.
	Digital transformation investments	*DI*	The level of investment in digital transformation
Independent variable	Sharing auditors with clients	*Comaud*	*Comaud* takes the value of 1 if the supplier employs the same audit enterprise as at least one of its top five customers during the year, and 0 otherwise.
Control variables	Business size	*Size*	Total assets at the end of the period are expressed in natural logarithms
	Age of business	*Age*	Years of business establishment
	Gearing ration	*Lev*	Ratio of total liabilities at the end of the period to total assets at the end of the Period
	Return on assets	*Roa*	Ratio of net profit at the end of the period to total assets at the end of the period
	Operating cash flow	*Cfo*	Net cash flows from operating activities at the end of the period to total assets at the end of the period
	Shareholding ratio of top ten shareholders	*Top10*	Ratio of number of shares held by top 10 shareholders to the total number of shares
	The size of the accounting enterprise	*Big4*	Takes the value of 1 if the accounting enterprise is an international Big 4, and 0 otherwise.
	Audit opinion	*Opinion*	If the enterprise obtains a standard unqualified opinion, it takes the value of 1, otherwise it takes the value of 0

### 4.3 Baseline model

To test the impact of sharing auditors with customers on the digital transformation of suppliers, we establish the following model:

(1) $$\begin{array}{c} {DT}_{i,t}\left({DI}_{i,t}\right)=\alpha_{0}+\alpha_{1}{\mathrm{Comaud}}_{i,t}+\alpha_{2}{\mathrm{Controls}}_{i,t}\\ +\sum \mathrm{Year}+\sum \mathrm{Industry}+\sum \mathrm{Firm}+\varepsilon \end{array}$$

In model (1), *DT* and *DI* represent the degree of digital transformation measured from the two dimensions of digital transformation keyword frequency and digital transformation investment; *Comaud* means whether the supplier shares auditors with its customers in the current year; *Controls* denotes the control variable; ∑*Year* denotes the year effect; ∑*Industry* denotes the industry effect; ∑*Firm* indicates the control of the enterprise’s individual fixed effect; and *ε* is the model random error term.

## 5 Empirical analyzes

### 5.1 Descriptive statistics

Table 2 reports the descriptive statistics results of the variables. The minimum and maximum values of enterprise digital transformation measured by digital transformation keyword frequency (*DT*) are 0.000 and 5.147, respectively, and the average value is 1.071, indicating that digital transformation varies significantly between different enterprises. The minimum and maximum values of digital transformation investment (*DI*) are 0.000 and 0.815, respectively, and the average value is 0.156, indicating that there are obvious differences in the level of digital transformation investment among different enterprises, and the digital transformation investment level of sample enterprises is generally not high. The average value of *Comaud* is 0.100, which means that 10% of suppliers employ the same accounting enterprise with at least one major client.

**Table 2:** Descriptive statistics of variables.

Variables	Observation	Mean	Std. dev	Min	Median	Max
*DT*	1,642	1.071	1.405	0.000	0.000	5.147
*DI*	1,642	0.156	0.184	0.000	0.090	0.815
*Comaud*	1,642	0.100	0.300	0.000	0.000	1.000
*Size*	1,642	21.867	1.259	19.692	21.689	25.419
*Age*	1,642	15.457	5.834	3.000	15.000	31.000
*Lev*	1,642	0.409	0.221	0.039	0.398	0.974
*Roa*	1,642	0.039	0.063	−0.278	0.040	0.193
*Cfo*	1,642	0.038	0.067	−0.175	0.038	0.215
*Top10*	1,642	59.366	15.621	24.120	61.028	91.260
*Big4*	1,642	0.046	0.210	0.000	0.000	1.000
*Opinion*	1,642	0.031	0.174	0.000	0.000	1.000

### 5.2 Regression analysis

Table 3 reports the analysis results of sharing auditors with customers on the digital transformation of suppliers. Column (1) shows that when measuring the digital transformation of enterprises with the keyword frequency (*DT*) of digital transformation, the regression coefficient of *Comaud* is 0.265, which is significantly positive at the level of 1%, indicating that sharing auditors with customers can significantly promote the digital transformation of suppliers, and in an economic sense, sharing auditors with customers can promote the digital transformation of suppliers by 24.7%.^3^ Column (2) shows that when measuring the digital transformation of enterprises with digital transformation investment (*DI*), the regression coefficient of *Comaud* is 0.059, which is significantly positive at the level of 1%, and in an economic sense, sharing auditors with customers can promote the digital transformation of suppliers by 5.1%.^4^ The above findings indicate that both in terms of statistical significance and economic significance, sharing auditors with customers can significantly enhance suppliers’ digital transformation, and the regression results support the research hypotheses of this study.

**Table 3:** Regression results of sharing auditors with clients on the digital transformation of suppliers.

Variables	(1)	(2)
	*DT*	*DI*
*Comaud*	0.265^***^	0.059^***^
	(2.640)	(4.342)
*Size*	0.410^***^	−0.029^***^
	(5.575)	(−2.956)
*Age*	0.153	−0.006
	(1.480)	(−0.413)
*Lev*	−0.302	0.026
	(−1.159)	(0.738)
*Roa*	−0.484	−0.034
	(−0.990)	(−0.520)
*Cfo*	−0.086	0.060
	(−0.237)	(1.222)
*Top10*	−0.009^***^	−0.001
	(−2.865)	(−1.505)
*Big4*	−0.165	−0.073^**^
	(−0.722)	(−2.376)
*Opinion*	0.003	0.025
	(0.018)	(1.118)
*Cons*	−7.593^***^	0.738^***^
	(−3.940)	(2.829)
*Year*	Yes	Yes
*Industry*	Yes	Yes
*Firm*	Yes	Yes
N	1,642	1,642
Adj_R^2^	0.351	0.140
		
		

***, **, and * indicate significance at the level of 1, 5, and 10%, respectively. T statistics are enclosed in parentheses.

### 5.3 Heterogeneity research

#### 5.3.1 Subsample research according to the bargaining power of suppliers

To test Hypothesis 2, the customer concentration of suppliers is selected to measure the bargaining power of suppliers, and higher customer concentration means weaker bargaining power of suppliers, and the sample enterprises are grouped based on the industry-annual median of the customer concentration of suppliers, and the model (1) is grouped for testing. Table 4 reports the results of the grouping test based on the bargaining power of the suppliers. Columns (1) and (2) are the test results of measuring the digital transformation of enterprises by the keyword frequency (*DT*) of digital transformation. Column (1) shows that in the low supplier bargaining power group, the coefficient of *Comaud* is 0.422 and is significant at a 1% level. Column (2) shows that in the high supplier bargaining power group, the coefficient of *Comaud* is −0.029, which is insignificant. Similarly, columns (3) and (4) show that when the digital transformation of an enterprise is measured by digital transformation investment (*DI*), *Comaud*’s regression coefficient is significant among suppliers with low bargaining power. The test results in Table 4 show that the effect of sharing auditors with customers on the digital transformation of suppliers is stronger in suppliers with low bargaining power, and Hypothesis 2 is confirmed.

**Table 4:** Heterogeneity analysis based on the bargaining power of suppliers.

Variables	(1)	(2)	(3)	(4)
	Supplier firms with weak bargaining power	Supplier firms with strong bargaining power	Supplier firms with weak bargaining power	Supplier firms with strong bargaining power
	*DT*	*DT*	*DI*	*DI*
*Comaud*	0.422^***^	−0.029	0.059^***^	0.041
	(2.749)	(−0.132)	(2.711)	(1.390)
*Size*	0.344^***^	0.640^***^	−0.021	−0.084^***^
	(3.646)	(3.392)	(−1.562)	(−3.267)
*Age*	0.232^**^	0.164	−0.008	−0.021
	(2.073)	(0.569)	(−0.480)	(−0.536)
*Lev*	−0.628^*^	0.340	0.043	−0.137^*^
	(−1.831)	(0.628)	(0.888)	(−1.862)
*Roa*	−0.413	0.643	−0.012	0.129
	(−0.704)	(0.541)	(−0.147)	(0.795)
*Cfo*	−0.213	−0.352	0.030	−0.000
	(−0.457)	(−0.447)	(0.452)	(−0.005)
*Top10*	−0.016^***^	0.009	−0.001	−0.002^*^
	(−3.496)	(1.149)	(−0.973)	(−1.927)
*Big4*	0.574^*^	−0.271	−0.075^*^	−0.072
	(1.922)	(−0.563)	(−1.761)	(−1.093)
*Opinion*	0.061	0.732	0.030	0.039
	(0.331)	(1.274)	(1.160)	(0.502)
*cons*	−7.652^***^	−15.188^***^	0.456	2.331^***^
	(−3.410)	(−3.013)	(1.426)	(3.395)
*Year*	Yes	Yes	Yes	Yes
*Industry*	Yes	Yes	Yes	Yes
*Firm*	Yes	Yes	Yes	Yes
N	948	597	948	597
Adj_R^2^	0.376	0.364	0.209	0.149

***, **, and * indicate significance at the level of 1, 5, and 10%, respectively. T statistics are enclosed in parentheses.

#### 5.3.2 Subsample research according to the media attention of suppliers

To test Hypothesis 3, the total number of media reports of suppliers is used to measure media attention, and the sample enterprises are grouped based on the industry-annual median of the media attention of suppliers, and the grouping test is conducted for model (1). Table 5 shows the results of the grouping test based on media attention. Column (1) shows that in the group with low media attention, the coefficient of *Comaud* is 0.510 and is significant at a 5% level. Column (2) shows that in the high media attention group, the coefficient of *Comaud* is 0.041 and is insignificant. Similarly, columns (3) and (4) show that when the digital transformation of an enterprise is measured by digital transformation investment (*DI*), *Comaud*’s regression coefficient is significant among suppliers with low media attention. The results of Table 5 show that the role of sharing auditors with customers on the digital transformation of suppliers is more significant in suppliers with low media attention, which means that sharing auditors with customers is an effective alternative mechanism for other forms of external supervision, as evidenced by Hypothesis 3.

**Table 5:** Heterogeneity analysis based on media attention of vendor companies.

Variables	(1)	(2)	(3)	(4)
	Supplier firms with low media attention	Supplier firms with high media attention	Supplier firms with low media attention	Supplier firms with high media attention
	*DT*	*DT*	*DI*	*DI*
*Comaud*	0.510^**^	0.041	0.128^***^	0.071^***^
	(2.523)	(0.257)	(4.295)	(3.187)
*Size*	0.436^***^	0.407^***^	−0.025	−0.052^**^
	(4.240)	(2.610)	(−1.633)	(−2.374)
*Age*	0.225^**^	0.071	−0.004	−0.021
	(2.011)	(0.194)	(−0.240)	(−0.402)
*Lev*	−0.355	−0.950^*^	−0.046	0.074
	(−0.923)	(−1.718)	(−0.807)	(0.947)
*Roa*	−0.094	0.237	−0.084	0.108
	(−0.144)	(0.225)	(−0.865)	(0.728)
*Cfo*	−0.247	0.435	0.074	0.097
	(−0.489)	(0.617)	(0.995)	(0.978)
*Top10*	−0.016^***^	−0.011	−0.000	−0.003^***^
	(−3.334)	(−1.648)	(−0.440)	(−3.142)
*Big4*	−0.097	−0.121	−0.019	−0.099^**^
	(−0.265)	(−0.341)	(−0.347)	(−1.974)
*Opinion*	−0.137	0.561	0.072^**^	−0.002
	(−0.639)	(1.502)	(2.286)	(−0.033)
*cons*	−10.608^***^	−7.671	0.603	1.339^**^
	(−4.237)	(−1.590)	(1.632)	(1.970)
*Year*	Yes	Yes	Yes	Yes
*Industry*	Yes	Yes	Yes	Yes
*Firm*	Yes	Yes	Yes	Yes
N	959	657	959	657
Adj_R^2^	0.409	0.373	0.154	0.218

***, **, and * indicate significance at the level of 1, 5, and 10%, respectively. T statistics are enclosed in parentheses.

#### 5.3.3 Subsample research according to the auditors’ industry expertise

To test Hypothesis 4, auditor industry expertise is measured by dividing the total client operating revenue of accounting enterprises within an industry by the total operating revenue of all customers in the industry, and enterprises with auditor industry expertise higher than the industry-annual median are categorized into the high auditor industry expertise group and enterprises lower than the industry-annual median are categorized into the low auditor industry expertise group. The model (1) is tested for grouping. Table 6 reports the results of the grouping tests based on the auditor’s industry expertise. Columns (1) and (2) are the test results of measuring the digital transformation of enterprises by the keyword frequency (*DT*) of digital transformation. Column (1) shows that in the low auditor industry expertise group, the coefficient of *Comaud* is 0.307 and is insignificant. Column (2) shows that in the high auditor industry expertise group, the regression coefficient of *Comaud* is 0.409, which is significant at the 1% level. Similarly, columns (3) and (4) show that *Comaud*’s regression coefficient is significant among vendors with higher auditor industry expertise when measuring digital transformation in terms of digital transformation investment (*DI*). The test results in Table 6 show that the role of sharing auditors with customers in promoting the digital transformation of suppliers is more significant in enterprises with high auditor industry expertise, which is consistent with theoretical expectations, and Hypothesis 4 is confirmed.

**Table 6:** Heterogeneity analysis based on the auditor’s industry expertise.

Variables	(1)	(2)	(3)	(4)
	Auditor with low industry expertise	Auditor with high industry expertise	Auditor with low industry expertise	Auditor with high industry expertise
	*DT*	*DT*	*DI*	*DI*
*Comaud*	0.307	0.409^***^	0.024	0.124^***^
	(1.368)	(2.909)	(0.959)	(5.993)
*Size*	0.656^***^	0.310^***^	−0.076^***^	−0.009
	(4.423)	(3.228)	(−4.572)	(−0.625)
*Age*	0.438	−0.001	−0.001	−0.022
	(1.333)	(−0.004)	(−0.026)	(−1.092)
*Lev*	−0.559	−0.179	0.001	0.024
	(−1.114)	(−0.507)	(0.014)	(0.462)
*Roa*	0.667	−0.184	−0.215^*^	0.106
	(0.662)	(−0.288)	(−1.902)	(1.121)
*Cfo*	−1.006	−0.132	−0.130^*^	0.127^*^
	(−1.527)	(−0.257)	(−1.758)	(1.675)
*Top10*	−0.014^**^	−0.008^*^	0.000	−0.001
	(−2.119)	(−1.862)	(0.148)	(−0.985)
*Big4*	−0.644	0.064	−0.108^**^	−0.045
	(−1.540)	(0.212)	(−2.296)	(−0.993)
*Opinion*	0.257	−0.071	−0.013	0.018
	(0.765)	(−0.338)	(−0.357)	(0.588)
*cons*	−17.154^***^	−4.803^**^	1.807^***^	0.173
	(−3.999)	(−2.004)	(3.747)	(0.491)
*Year*	Yes	Yes	Yes	Yes
*Industry*	Yes	Yes	Yes	Yes
*Firm*	Yes	Yes	Yes	Yes
N	657	985	657	985
Adj_R^2^	0.391	0.353	0.244	0.202

***, **, and * indicate significance at the level of 1, 5, and 10%, respectively. T statistics are enclosed in parentheses.

### 5.4 Endogenous test

#### 5.4.1 Placebo test

**Figure 2:**
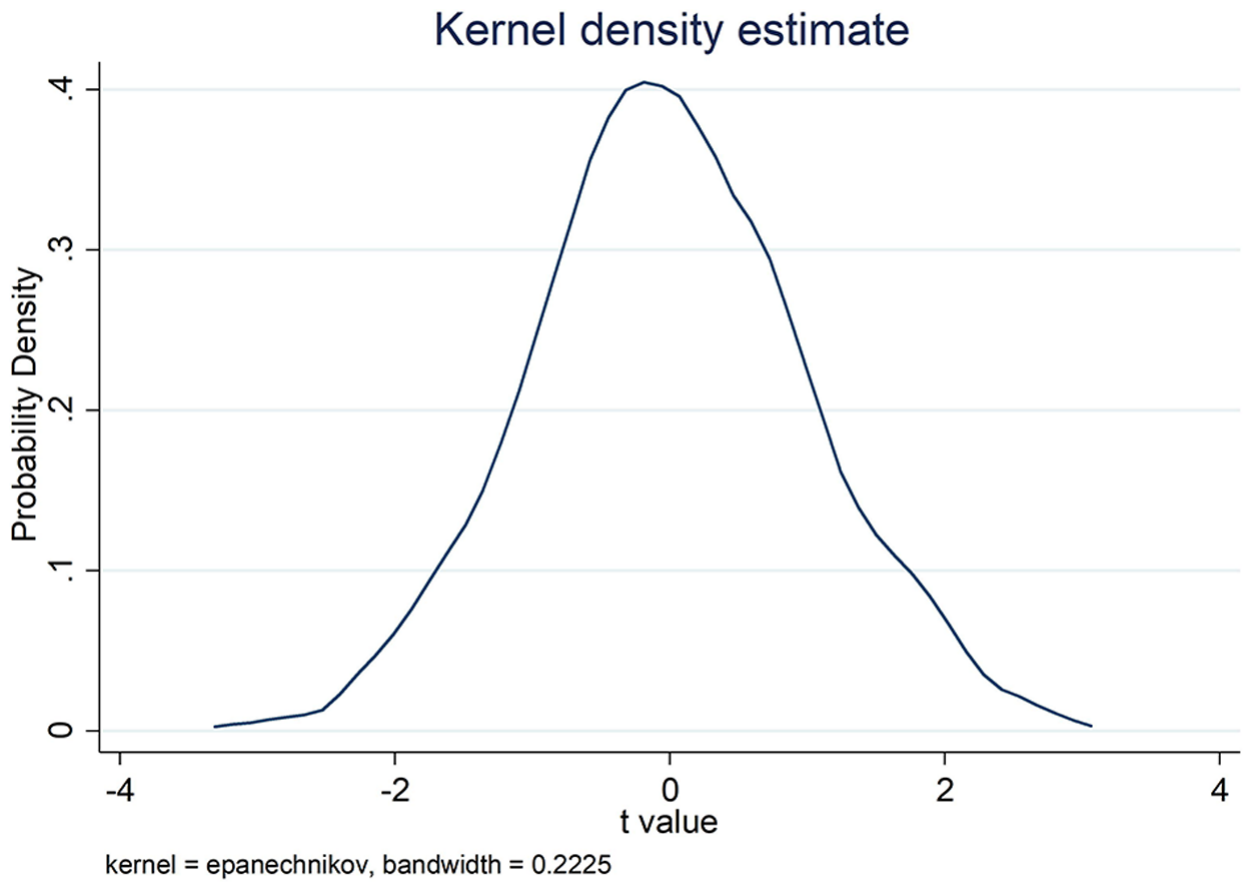
Results of placebo test with *DT* as the dependent variable.

**Figure 3:**
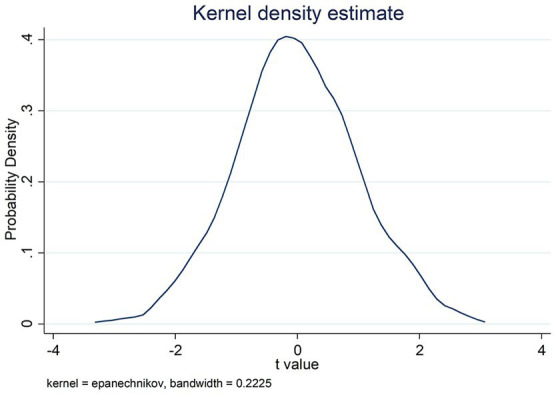
Results of placebo test with *DI* as the dependent variable.

To exclude the interference of omitted variables on the findings of the study, the placebo test (Placebo test) was used for the robustness test. The specific idea is to randomly designate suppliers to share auditors with customers according to the proportion of shared auditors in the original sample, accordingly, conduct regression analysis and repeat it 1,000 times to obtain the T-value distribution of regression coefficients of shared auditors with customers as shown in Figure 2 (*DT* as a measure of enterprise digital transformation) and Figure 3 (as a measure of enterprise digital transformation as *DI*). Figures 2, 3 report that the T-value of the regression coefficient of the randomly designated shared auditor with customers is concentrated around 0, basically obeying a normal distribution, which excludes that the findings of this study are caused by unobservable factors and proves that the conclusions obtained in the previous study are robust.

#### 5.4.2 PSM model

To mitigate the possible reverse causation problem, the PSM (Propensity Score Matching) method is used for robustness testing. First, using the control variables as covariates, whether suppliers share auditors with customers as independent variables and digital transformation of suppliers (*DT* and *DI*) as dependent variables, the Logit model is used to perform nearest-neighbor matching in the ratio of 1:3, and the T-value of the gap between the two groups of samples on whether there is a shared auditor or not after the matching (ATT) becomes smaller, which indicates that the matching effect is good. Second, regression is conducted again based on the matched samples, and the results can be seen in column (1) and column (2) of Table 7; the coefficients of *Comaud* are significant at a 1% level, indicating that the regression results in the previous article are robust.

**Table 7:** PSM test and Heckman’s two-stage regression.

Variables	(1)	(2)	(3)	(4)
	PSM		Heckman’s two stages – phase II	
	*DT*	*DI*	*DT*	*DI*
*Comaud*	0.426^***^	0.101^***^	0.229^**^	0.065^***^
	(3.485)	(4.126)	(2.160)	(4.379)
*Size*	0.829^***^	−0.064^*^	0.314^***^	−0.031^**^
	(4.809)	(−1.862)	(3.310)	(−2.306)
*Age*	0.259	−0.054	0.208^*^	−0.009
	(0.672)	(−0.698)	(1.928)	(−0.604)
*Lev*	−1.064	−0.111	−0.148	0.003
	(−1.605)	(−0.836)	(−0.499)	(0.068)
*Roa*	−0.095	−0.066	0.134	−0.047
	(−0.077)	(−0.268)	(0.238)	(−0.589)
*Cfo*	−0.273	0.261	−0.248	0.081
	(−0.306)	(1.464)	(−0.591)	(1.373)
*Top10*	−0.009	−0.003^*^	−0.008^**^	−0.001^**^
	(−1.269)	(−1.802)	(−2.159)	(−2.095)
*Big4*	−0.251	−0.110^**^	−1.393^**^	−0.200^**^
	(−1.011)	(−2.218)	(−2.014)	(−2.064)
*Opinion*	0.196	0.013	0.537^*^	0.055
	(0.530)	(0.174)	(1.730)	(1.266)
*Imr*			−1.344	−0.141
			(−1.633)	(−1.220)
*cons*	−17.956^***^	1.925^*^	−5.460^**^	0.846^**^
	(−3.450)	(1.849)	(−1.993)	(2.200)
*Year*	Yes	Yes	Yes	Yes
*Industry*	Yes	Yes	Yes	Yes
*Firm*	Yes	Yes	Yes	Yes
N	529	529	1,339	1,339
Adj_R^2^	0.448	0.299	0.331	0.129

***, **, and * indicate significance at the level of 1, 5, and 10%, respectively. T statistics are enclosed in parentheses.

#### 5.4.3 Heckman’s two-stage regression

To alleviate the sample self-selection problem, Heckman’s two-stage method is used for robustness testing. In the first stage, audit fee (*Fee*), whether the auditor is an international Big 4 (*Big4*), and audit opinion (*Opinion*) are selected as the influencing factors of whether suppliers share auditors with their customers, and the Probit model is applied to calculate the inverse Mills ratio (*Imr*). In the second stage, we add the inverse Mills ratio (*Imr*) to the main regression model (1) to obtain the second stage regression model (2):

(2) $$\begin{array}{c} {DT}_{i,t}\left({DI}_{i,t}\right)=\alpha_{0}+\alpha_{1}{\mathrm{Comaud}}_{i,t}+\alpha_{2}{Imr}_{i,t}+\alpha_{3}{\mathrm{Controls}}_{i,t}\\ +\sum \mathrm{Year}+\sum \mathrm{Industry}+\sum \mathrm{Firm}+\varepsilon \end{array}$$

In column (3) and column (4) of Table 7, the coefficients of *Comaud* are both significant after controlling for the inverse *Imr*, which once again proved the robustness of the regression results in this study.

### 5.5 Robustness tests

#### 5.5.1 Excluding the impact of financial shocks

Considering the close relationship between the development of the digital economy and the overall financial dynamics, ignoring such factors may generate estimation bias. There are two financial event shocks, the 2008 international financial crisis and the 2015 Chinese stock market crash, in the time series of this study; thus, the research samples of 2008 and 2015 are excluded from model (1) and column (1) and column (2) of Table 8 show that the conclusion is still robust.

**Table 8:** Robustness tests.

Variables	(1)	(2)	(3)	(4)
	Excluding the impact of financial shocks		Control for higher-order fixed effects	
	*DT*	*DI*	*DT*	*DI*
*Comaud*	0.256^***^	0.054^***^	0.252^**^	0.080^***^
	(2.619)	(3.889)	(2.114)	(5.198)
*Size*	0.419^***^	−0.027^**^	0.488^***^	−0.031^***^
	(5.421)	(−2.409)	(5.292)	(−2.610)
*Age*	0.255	−0.010	0.247^**^	−0.006
	(1.552)	(−0.419)	(2.112)	(−0.397)
*Lev*	−0.315	0.007	−0.511	−0.033
	(−1.161)	(0.178)	(−1.565)	(−0.775)
*Roa*	−0.335	−0.047	0.028	−0.072
	(−0.654)	(−0.645)	(0.046)	(−0.920)
*Cfo*	−0.437	0.035	−0.013	0.019
	(−1.204)	(0.675)	(−0.030)	(0.338)
*Top10*	−0.011^***^	−0.001^*^	−0.005	0.000
	(−3.190)	(−1.805)	(−1.080)	(0.147)
*Big4*	−0.213	−0.089^***^	0.027	−0.082^**^
	(−0.976)	(−2.881)	(0.094)	(−2.212)
*Opinion*	0.224	0.023	0.145	0.018
	(1.335)	(0.953)	(0.712)	(0.668)
*cons*	−8.619^***^	0.794^**^	−12.927^***^	0.933^***^
	(−3.711)	(2.402)	(−4.802)	(2.672)
*Year*	Yes	Yes	Yes	Yes
*Industry*	Yes	Yes	Yes	Yes
*Firm*	Yes	Yes	Yes	Yes
*Year×Province*	No	No	Yes	Yes
N	1,509	1,509	1,182	1,182
Adj_R^2^	0.387	0.150	0.922	0.916

***, **, and * indicate significance at the level of 1, 5, and 10%, respectively. T statistics are enclosed in parentheses.

#### 5.5.2 Controlling for more fixed effects

To avoid the interference of unobservable factors at the regional level over time, the high-order fixed effects of “*Year×Provinces*” were further controlled based on the benchmark assumption to control the time-fixed effect, the industry fixed effect, and the individual fixed effect, and the regression results are shown in columns (3) and (4) in Table 8. The results of the above robustness test are consistent with the benchmark regression results.

## 6 Influence mechanism

The above results prove that sharing auditors with customers can promote the digital transformation of suppliers. According to the previous theoretical analysis, the facilitation effect of sharing auditors with customers is mainly realized through two paths: strengthening the supervision of suppliers and alleviating the financing constraints of suppliers. To further verify the previous theoretical analysis, model (3) is constructed. In model (3), M denotes the degree of supervision of suppliers (*Ac*) and the degree of supplier financing constraints (*KZ*). In the calculation of the supervision of suppliers, the ratio of management expenses to operating income is used to measure, and the larger the value of *Ac*, the smaller the supervision of suppliers. In the calculation of financing constraints, referring to the study of Kaplan and Zingales (1997), the KZ index is used to measure the degree of financing constraints, and the larger the KZ index is, the higher the degree of financing constraints of the supplier.

(3) $$\begin{array}{c} M_{i,t}=\alpha_{0}+\alpha_{1}{\mathrm{Comaud}}_{i,t}+\alpha_{2}{\mathrm{Controls}}_{i,t}\\ +\sum \mathrm{Year}+\sum \mathrm{Industry}+\sum \mathrm{Firm}+\varepsilon \end{array}$$

Table 9 shows the results of the mechanism test. Columns (1) and (2) show that sharing auditors with customers significantly facilitates the digital transformation of suppliers. Column (3) shows that the regression coefficient between the supervision of *Comaud* and the supervision of the supplier is −0.013, which is significant at the 5% level, indicating that the auditor who shares with the client can significantly enhance the supervision of the supplier. Column (6) shows that the regression coefficient between the financing constraints of *Comaud* is −0.327, which is significant at the 10% level, indicating that the sharing of the auditors with the customers can significantly alleviate the financing constraints of the suppliers. Table 9 verifies that sharing auditors with customers can promote the digital transformation of suppliers by enhancing the supervision of suppliers and easing financing constraints.

**Table 9:** Influence mechanism test.

Variables	(1)	(2)	(3)	(4)	(5)	(6)
	*DT*	*DI*	*Ac*	*DT*	*DI*	*KZ*
*Comaud*	0.265^***^	0.059^***^	−0.013^**^	0.242^**^	0.057^***^	−0.327^*^
	(2.640)	(4.342)	(−2.064)	(2.314)	(3.929)	(−1.888)
*Size*	0.410^***^	−0.029^***^	−0.039^***^	0.478^***^	−0.044^***^	−1.156^***^
	(5.575)	(−2.956)	(−8.385)	(5.569)	(−3.641)	(−8.122)
*Age*	0.153	−0.006	0.003	0.208^*^	0.000	−0.055
	(1.480)	(−0.413)	(0.427)	(1.806)	(0.006)	(−0.287)
*Lev*	−0.302	0.026	0.039^**^	−0.403	0.030	7.971^***^
	(−1.159)	(0.738)	(2.389)	(−1.346)	(0.726)	(16.065)
*Roa*	−0.484	−0.034	−0.170^***^	−0.919	−0.020	−1.844^*^
	(−0.990)	(−0.520)	(−5.540)	(−1.584)	(−0.249)	(−1.918)
*Cfo*	−0.086	0.060	0.031	0.431	0.066	−14.616^***^
	(−0.237)	(1.222)	(1.352)	(1.040)	(1.140)	(−21.288)
*Top10*	−0.009^***^	−0.001	0.000	−0.009^**^	−0.001^**^	−0.025^***^
	(−2.865)	(−1.505)	(1.338)	(−2.353)	(−1.982)	(−4.114)
*Big4*	−0.165	−0.073^**^	0.009	−0.152	−0.044	−0.327
	(−0.722)	(−2.376)	(0.643)	(−0.601)	(−1.240)	(−0.780)
*Opinion*	0.003	0.025	0.029^***^	−0.118	0.007	−0.447
	(0.018)	(1.118)	(2.779)	(−0.625)	(0.270)	(−1.432)
*cons*	−7.593^***^	0.738^***^	0.852^***^	−9.723^***^	1.044^***^	27.176^***^
	(−3.940)	(2.829)	(7.044)	(−4.473)	(3.431)	(7.544)
*Year*	Yes	Yes	Yes	Yes	Yes	Yes
*Industry*	Yes	Yes	Yes	Yes	Yes	Yes
*Firm*	Yes	Yes	Yes	Yes	Yes	Yes
N	1,642	1,642	1,642	1,403	1,403	1,403
Adj_R^2^	0.351	0.140	0.253	0.351	0.127	0.653

***, **, and * indicate significance at the level of 1, 5, and 10%, respectively. T statistics are enclosed in parentheses.

## 7 Further discussion

### 7.1 Discussion of the alternative hypothesis

According to the theory of behavioral economics, out of the aversion to loss and the desire to continue their careers, managers are unable to focus on the long-term sustainable development of the enterprise, resulting in managers being prone to short-sightedness when making decisions on inter-period choices, which leads to the tendency of managers to avoid long-cycle and high-risk digital transformation projects (Wang et al., 2024). Thus, the digital transformation of suppliers may also be determined by the investment horizons of the enterprises’ managers. To exclude the alternative hypothesis that the digital transformation of suppliers is due to managers’ investment horizons, the short-sightedness of managers in suppliers is further added as a control variable based on model (1), and this study adopts the total word frequency of managers’ “short-term horizons” vocabulary (*Myopia_Index*) and the total words frequency of corporate stock exchange (*Myopia_Index*) in MD&A, respectively. In this study, the total word frequency of managers’ “short-term horizon” words in MD&A (*Myopia_Index*) and *stockturnover* are used to measure managers’ short-sightedness. Table 10 indicates that the coefficient on sharing auditors with customers remains significantly positive after controlling for the investment horizons of managers of suppliers, which excludes alternative explanations for management’s investment horizon.

**Table 10:** Alternative explanations test.

Variables	(1)	(2)	(3)	(4)
	*DT*	*DI*	*DT*	*DI*
*Comaud*	0.267^***^	0.059^***^	0.267^***^	0.059^***^
	(2.661)	(4.352)	(2.658)	(4.344)
*Myopia_Index*	−0.003	−0.000		
	(−0.957)	(−0.569)		
*Stockturnover*			0.013	−0.002
			(0.646)	(−0.863)
*Size*	0.421^***^	−0.029^***^	0.456^***^	−0.039^***^
	(5.656)	(−2.830)	(5.900)	(−3.757)
*Age*	0.156	−0.006	0.156	−0.006
	(1.507)	(−0.396)	(1.509)	(−0.424)
*Lev*	−0.277	0.028	−0.432	0.052
	(−1.059)	(0.790)	(−1.610)	(1.432)
*Roa*	−0.483	−0.034	−0.366	−0.054
	(−0.989)	(−0.519)	(−0.725)	(−0.798)
*Cfo*	−0.086	0.060	−0.010	0.051
	(−0.237)	(1.222)	(−0.028)	(1.032)
*Top10*	−0.009^***^	−0.001	−0.008^**^	−0.001^*^
	(−2.802)	(−1.467)	(−2.403)	(−1.815)
*Big4*	−0.150	−0.072^**^	−0.169	−0.073^**^
	(−0.654)	(−2.330)	(−0.736)	(−2.369)
*Opinion*	0.018	0.026	0.068	0.005
	(0.112)	(1.169)	(0.400)	(0.216)
*cons*	−7.824^***^	0.720^***^	−8.620^***^	0.944^***^
	(−4.029)	(2.736)	(−4.284)	(3.477)
*Year*	Yes	Yes	Yes	Yes
*Industry*	Yes	Yes	Yes	Yes
*Firm*	Yes	Yes	Yes	Yes
N	1,642	1,642	1,636	1,636
Adj_R^2^	0.352	0.140	0.354	0.146

***, **, and * indicate significance at the level of 1, 5, and 10%, respectively. T statistics are enclosed in parentheses.

### 7.2 Economic consequences test

As a cutting-edge transformation mode in the new era, digital transformation can empower suppliers’ innovation activities. On the one hand, enterprise digital transformation releases a favorable signal for enterprises to actively respond to national policies, and the enterprise is more likely to be trusted and sought after in the capital market, which helps to alleviate the pressure on the capital of innovation activities (Wu et al., 2021). On the other hand, digital transformation can accelerate the low-cost penetration of information, which helps enterprises accurately grasp the changes in market demand with the help of advanced digital technology, significantly reduces the uncertainty in the process of R&D and innovation, lowers the cost of trial and error, and facilitates the increase of innovation output. Furthermore, with the gradual deepening of the digital transformation process, the operational efficiency of enterprises has been significantly improved, and enterprises can achieve higher marginal innovation output under the original resource boundary (Wu et al., 2021). When suppliers share auditors with their customers, the auditors reduce the uncertainty of R&D and innovation activities by improving the information environment of suppliers, which further promotes suppliers’ innovation. Based on the above analysis, model (4) is constructed to examine whether digital transformation helps to promote suppliers’ innovation in suppliers who share auditors with customers.

(4) $$\begin{array}{c} \left({\mathrm{Patent}}_{i,t+1}\right)=\alpha_{0}+\alpha_{1}{DT}_{i,t}\left({DI}_{i,t}\right)+\alpha_{2}{\mathrm{Comaud}}_{i,t}\\ +\;\alpha_{3}{DT}_{i,t}\left({DI}_{i,t}\right)\times {\mathrm{Comaud}}_{i,t}+\;\alpha_{4}{\mathrm{Controls}}_{i,t}\\ +\sum \mathrm{Year}+\sum \mathrm{Industry}+\sum \mathrm{Firm}+\varepsilon \end{array}$$

In model (4), *Patent_i,t + 1_* is the level of innovation of suppliers in the next year, measured by the number of enterprise invention patent applications. Table 11 shows that when digital transformation is measured by digital transformation keyword word frequency (*DT*), the regression coefficients of the cross-multiple terms *Comaud* × *DT* are both significantly positive. Column (2) shows that when the digital transformation investment (*DI*) is used to measure the digital transformation of enterprises, the regression coefficients of the *Comaud×DI* terms are also significantly positive. The test results in Table 11 show that when suppliers share auditors with customers, the digital transformation of suppliers can promote their innovation level.

**Table 11:** Economic consequences test.

Variables	(1)	(2)
	Patent	Patent
*Comaud×DT*	3.971^**^	
	(2.111)	
*Comaud×DI*		45.548^*^
		(1.931)
*Comaud*	−8.160^**^	−9.000^**^
	(−2.321)	(−2.363)
*DT*	−2.475^***^	
	(−2.706)	
*DI*		−1.179
		(−0.143)
*Size*	2.541	1.736
	(1.312)	(0.905)
*Age*	−1.546	−1.484
	(−0.317)	(−0.302)
*Lev*	0.275	−1.793
	(0.038)	(−0.244)
*Roa*	9.519	8.662
	(0.769)	(0.692)
*Cfo*	−2.183	−2.997
	(−0.248)	(−0.336)
*Top10*	−0.323^***^	−0.316^***^
	(−3.844)	(−3.743)
*Big4*	3.616	4.632
	(0.713)	(0.904)
*Opinion*	12.879^***^	12.771^***^
	(3.274)	(3.216)
*Cons*	−7.224	3.939
	(−0.122)	(0.066)
*Year*	Yes	Yes
*Industry*	Yes	Yes
*Firm*	Yes	Yes
N	725	725
Adj_R^2^	0.218	0.205

***, **, and * indicate significance at the level of 1, 5, and 10%, respectively. T statistics are enclosed in parentheses.

## 8 Conclusion and implications

This study examines the impact of sharing auditors with customers on the digital transformation of suppliers. It is found that sharing auditors with customers helps promote suppliers’ digital transformation, and this facilitating effect is stronger among suppliers with weaker bargaining power, lower media attention, and higher auditor industry expertise. The results of the mechanism of action test indicate that sharing auditors with customers promotes suppliers’ digital transformation by strengthening the supervision of the supplier and alleviating the financing constraints of the supplier. Further research finds that when suppliers share auditors with their customers, suppliers’ digital transformation helps to promote their innovation.

The findings of this study have important policy value. First, enterprises should consider hiring the same auditor as their major customers to take advantage of sharing auditor information and help enterprises in digital transformation. Auditors are not only financial statement certifiers but also information intermediaries with rich knowledge accumulation and abundant information resources. Enterprises should attach importance to the important role of auditors and actively explore and utilize the social network resources of shared auditors to maximize the digital transformation promotion effect of shared auditors and the alternative effect of external supervision mechanisms to provide external impetus for alleviating the problems of digital transformation of enterprises and promoting the development of the digital economy. Second, for auditors, they should change their competitive strategy and implement a quality-oriented competitive strategy. Auditors should abandon the traditional behavior of “low price solicitation,” strive to improve their own professional level and industry expertise, focus on strengthening internal construction oriented to improving audit quality, and maximize the use of “signal transmission” and “information sharing” of shared auditors. Shared auditors can maximize the functions of “signal transmission” and “information sharing” to help enterprises implement digital transformation strategies and innovation strategies, thereby promoting enterprises to move toward the goal of high-quality development. Third, government departments should appropriately guide establishing a shared auditor system between suppliers and their customers to promote the digital transformation of enterprises in an orderly manner. Relevant government departments can establish a shared auditor system, for example, to give certain incentives to suppliers that share auditors with customers and enhance the positive role of shared auditors during the enterprise’s digital transformation through system supply. This study finds that sharing auditors with customers has a stronger facilitating effect on the digital transformation of suppliers with lower media attention, indicating that shared auditors can serve as an effective alternative to external monitoring mechanisms, and thus supply chain-shared auditors can be regarded as a new initiative for the government to monitor enterprises. In addition, considering the problems in the process of digital transformation, such as the difficulty in ensuring the security of enterprise data and the leakage of sensitive information, the government should strengthen supervision and introduce corresponding policies and laws to regulate and solve the worries of enterprises in digital transformation.

## Data availability statement

The raw data supporting the conclusions of this article will be made available by the authors, without undue reservation.

## Author contributions

XHL: Writing – original draft, Writing – review & editing. YC: Supervision, Writing – review & editing.

## Footnotes

^1^China Academy of Information and Communications Technology: White Paper on Cloud Computing Development (2018), https://max.book118.com/html/2018/0815/7,065,042,041,001,143.shtm.

^2^“Will not transform” refers to insufficient digital transformation capabilities; “dare not transform” refers to the long pain period of digital transformation.

^3^In Table 3, the ratio between the regression coefficient of *Comaud* (0.265) and the mean of *DT* (1.071) is 0.247.

^4^In Table 3, the ratio between the regression coefficient of *Comaud* (0.059) and the mean of *DI* (0.156) in column (2) is 0.051.
